# Arsenic redox transformations and cycling in the rhizosphere of *Pteris vittata* and *Pteris quadriaurita*

**DOI:** 10.1016/j.envexpbot.2020.104122

**Published:** 2020-05-20

**Authors:** Stefan Wagner, Christoph Hoefer, Markus Puschenreiter, Walter W. Wenzel, Eva Oburger, Stephan Hann, Brett Robinson, Ruben Kretzschmar, Jakob Santner

**Affiliations:** a Department of Forest and Soil Sciences, Institute of Soil Research, Rhizosphere Ecology & Biogeochemistry Group, University of Natural Resources and Life Sciences, Vienna, Konrad-Lorenz-Strasse 24, 3430, Tulln, Austria; b Department General, Analytical and Physical Chemistry, Chair of General and Analytical Chemistry, Montanuniversität Leoben, Franz-Josef-Strasse 18, 8700, Leoben, Austria; c Department of Chemistry, Institute of Analytical Chemistry, University of Natural Resources and Life Sciences, Vienna, Muthgasse 18, 1190, Vienna, Austria; d Department of Environmental Systems Science, Institute of Biogeochemistry and Pollutant Dynamics, Soil Chemistry Group, ETH Zürich, Universitätstrasse 16, CHN, 8092, Zürich, Switzerland; e School of Physical and Chemical Sciences, University of Canterbury, 20 Kirkwood Ave, Ilam, Christchurch, 8041, New Zealand; f Department of Crop Sciences, Institute of Agronomy, University of Natural Resources and Life Sciences, Vienna, Konrad-Lorenz-Strasse 24, 3430, Tulln, Austria

**Keywords:** Chemical imaging, Arsenic speciation, Rhizosphere, Diffusive gradients in thin films, Laser ablation inductively coupled plasma mass spectrometry, Planar optodes

## Abstract

*Pteris vittata* (PV) and *Pteris quadriaurita* (PQ) are reported to hyperaccumulate arsenic (As) when grown in Asrich soil. Yet, little is known about the impact of their unique As accumulation mechanisms on As transformations and cycling at the soil-root interface. Using a combined approach of two-dimensional (2D), sub-mm scale solute imaging of arsenite (As^III^), arsenate (As^V^), phosphorus (P), manganese (Mn), iron (Fe) and oxygen (O_2_), we found localized patterns of As^III^/As^V^ redox transformations in the PV rhizosphere (As^III^/As^V^ ratio of 0.57) compared to bulk soil (As^III^/As^V^ ratio of ≤0.04). Our data indicate that the high As root uptake, translocation and accumulation from the As-rich experimental soil (2080 mg kg^-1^) to PV fronds (6986 mg kg^-1^) induced As detoxification via As^V^ reduction and As^III^ root efflux, leading to As^III^ accumulation and re-oxidation to As^V^ in the rhizosphere porewater. This As cycling mechanism is linked to the reduction of O2 and Mn^III/IV^ (oxyhydr)oxides resulting in decreased O2 levels and increased Mn solubilization along roots. Compared to PV, we found 4-fold lower As translocation to PQ fronds (1611 mg kg^-1^), 2-fold lower As^V^ depletion in the PQ rhizosphere, and no As^III^ efflux from PQ roots, suggesting that PQ efficiently controls As uptake to avoid toxic As levels in roots. Analysis of root exudates obtained from soil-grown PV showed that As acquisition by PV roots was not associated with phytic acid release. Our study demonstrates that two closely-related As-accumulating ferns have distinct mechanisms for As uptake modulating As cycling in As-rich environments.

## 1 Introduction

Arsenic (As) is a widespread and persistent metalloid contaminant in natural soil environments. From As-rich (>10 mg kg^-1^) soil, As can be taken up by plants, may enter food and fodder, or leach into ground- and surface waters, causing severe environmental and human health problems (Zhao et al., 2009, 2010; Wenzel, 2013; Zhao et al., 2015). Terrestrial plants generally restrict As uptake in roots and translocation to shoots to survive As stress (Fitz and Wenzel, 2006). In contrast, 13 species from the *Pteris* genus of ferns, including *Pteris vittata* (PV) and *Pteris quadriaurita* (PQ), are known to hyperaccumulate As (Chen et al., 2018; Claveria et al., 2019), showing efficient As root uptake and translocation from As-rich soil to aboveground tissues (i.e. fronds) (Ma et al., 2001; Zhao et al., 2002; Meharg, 2003; Srivastava et al., 2006), which is used for the plant-based remediation (i.e. phytoremediation) of As-contaminated environments (Tu et al., 2004a; Wang and Ma, 2015; da Silva et al., 2018).

The mobility of As in soil and thus its availability to plants is determined by its speciation (Fitz and Wenzel, 2002, 2006; Zhao et al., 2009; Wenzel, 2013). Hyperaccumulator ferns are native to aerobic (oxic) soil environments (Jones, 1987), where arsenate (As^V^) is the predominant As species in soil solution. Under anaerobic (anoxic) conditions, abiotic reduction of As^V^ to arsenite (As^III^) can increase As mobility in soil, because As^III^ is more weakly bound to most soil minerals compared to As^V^ (Wenzel, 2013). Organic As species are typically present only in minor proportions in most mineral soils (Huang et al., 2011a). Plant uptake of As^V^ and As^III^ proceeds inadvertently via transporter systems generally responsible for phosphate (P) and silicon (Si) uptake (Zhao et al., 2009). Redox-sensitive elements, such as iron (Fe) or manganese (Mn), often interact with As in soil and their relative abundance and speciation in the rhizosphere determine the fate of As in the environment (Borch et al., 2010). For example, reductive dissolution of Fe^III^ (oxyhydr)oxides to soluble Fe^II^ following prolonged anoxia in e.g. paddy soils can result in concomitant release and reduction of adsorbed As^V^, liberating As^III^ into solution (Takahashi et al., 2004). Conversely, Mn^III^ ^/IV^ (oxyhydr)oxides can reduce As mobility by mediating As^III^ oxidation to As^V^, enhancing As adsorption on Fe^III^ (oxyhydr) oxide surfaces (Oscarson et al., 1981; Ying et al., 2012).

Hyperaccumulator roots have evolved unique mechanisms that can potentially change As speciation and mobility in soil (Fitz and Wenzel, 2002; Xie et al., 2009; Han et al., 2017). Previous work where PV was grown in hydroponics indicated that PV may actively release phytic acid from its roots to mobilize As^V^ from sparingly soluble mineral surfaces by competitive desorption (Tu et al., 2004b; Liu et al., 2016). Although Liu et al. (2016) proposed that this represents an important mechanism of hyperaccumulator ferns for enhanced As accumulation, verification of phytic acid exudation under soil-based conditions is still missing. Inside PV roots, As^V^ is rapidly reduced to As^III^ via As^V^ reductase (Duan et al., 2005; Liu et al., 2009; Cesaro et al., 2015). Subsequently, a major As^III^ fraction is efficiently translocated into frond tissues via xylem transport (Su et al., 2008) and sequestered both intracellularly in the epidermal cell vacuoles (Lombi et al., 2002) and extracellularly in the pinnae cell apoplasm (Datta et al., 2017). However, a fraction of the internal As^III^ may be released (effluxed) from root tissues into the external medium (Su et al., 2008; Huang et al., 2011b; Chen et al., 2016; Han et al., 2016). For example, Chen et al. (2016) found rapid As^V^ reduction to As^III^ in sterile nutrient solution culture where PV roots were exposed to As^V^ concentrations of ≥ 15 mg L^-1^. This is consistent with a mechanism of As^III^ root efflux for As detoxification, which is reported for several non-accumulating plants such as *Oryza sativa, Lycopersicon esculentum* and *Zea mays* (Xu et al., 2007; Zhao et al., 2009). Chen et al. (2016) demonstrated that the change in As speciation was a direct result of As^III^ efflux and not due to As transformation by microbes or root exudates. However, the high As^V^ concentrations (≥15mg L^-1^) in the hydroponic solution used in this study exceeded As porewater concentrations found in even highly As contaminated soils (Wenzel et al., 2002). Moreover, hydroponics are not directly comparable with soil-based systems (Oburger and Jones, 2018), and do not allow to investigate As redox transformations and interactions with other elements in the rhizosphere at relevant spatial scales.

The As speciation and flux dynamics in the rhizosphere environment of soil-grown hyperaccumulator ferns is largely unknown. In an earlier study on PV rhizosphere characteristics, Fitz et al. (2003) used a rhizobox experiment with a rhizosphere slicing technique to resolve onedimensional As gradients parallel to a root mat at ~ 1 mm spatial resolution. Sampling of the labile (i.e. reversibly adsorbed) As fraction by diffusive gradients in thin films (DGT) (Davison and Zhang, 1994) revealed substantial depletion of labile As extending up to ~ 3 mm from the root mat into the rhizosphere soil. In the rhizosphere porewater, however, no changes in the inorganic As concentration and speciation were measured throughout the growth period. The slightly decreased soil redox potential and increased Fe concentrations in the rhizosphere porewater compared to bulk soil indicated reductive co-dissolution of As^V^ from Fe^III^ (oxyhydr)oxides, thus sustaining As^V^ concentrations in the rhizosphere. Advanced as the experimental approach of Fitz et al. (2003) was at its time, it could only partially explain the observed As resupply, since measurements were performed *ex situ* and at inadequate spatial resolution to detect potential As transformation and mobilization processes in the rhizosphere microenvironment.

An alternative approach is to use rhizotron experiments in combination with solute imaging techniques such as DGT coupled to laser ablation inductively coupled plasma mass spectrometry (LA-ICP-MS) and/or planar optodes (Santner et al., 2015; Oburger and Schmidt, 2016; Santner and Williams, 2016). These techniques combine passive solute sampling with high-resolution chemical imaging methods, allowing for simultaneous, sub-mm scale mapping of multi-elemental solute flux distributions across the two-dimensional (2D) soil-root interface. For example, the combination of DGT with LA-ICP-MS was used to study Cd and Zn mobilization after S° addition in the rhizosphere of metal-accumulating *Salix smithiana* (Hoefer et al., 2015), while DGT and planar optode imaging was used to study pH-dependent P and Fe solubilization processes in the rhizosphere of the seagrass *Cymodocea serrulata* (Brodersen et al., 2017).

We aimed to investigate the distribution of As^III^ and As^V^ in the rhizosphere of the two As hyperaccumulator ferns *P. vittata* and *P. quadriaurita* grown in soil geogenically enriched in As. In addition, we measured the distribution of Mn, Fe, P and O_2_, which are potentially involved in the redox transformation and cycling of As. In line with previous evidence, we hypothesized that (1) As is mobilized in the rhizosphere due to reductive co-dissolution of As^V^/As^III^ from Fe^III^ (oxyhydr)oxides (Fitz et al., 2003), (2) As^III^ is effluxed from fern roots into the rhizosphere after As^V^ reduction to As^III^ inside the roots (Su et al., 2008; Huang et al., 2011b; Chen et al., 2016; Han et al., 2016), and (3) As accumulation by PV is linked to phytic acid exudation from roots (Tu et al., 2004b; Liu et al., 2016). To this end, we mapped labile As^III^, As^V^, P, Mn and Fe along individual PV and PQ roots using a combined dual-layer DGT LA-ICP-MS technique in a rhizotron setup. In parallel, we applied planar optodes to map the distribution of O_2_ as an indicator for the redox status in rooted soil of PV and PQ. Accumulation of total As in fern frond and root tissues was assessed by biomass digestion and ICP-MS analysis. Moreover, root exudates of soil-grown ferns were analyzed for phytic acid using a rhizobox setup and ion chromatography combined with ICP-MS.

## 2 Materials and methods

### 2.1 Experimental soil

The experimental soil, an upper B horizon of a Calcaric Cambisol (IUSS Working Group WRB, 2015), was collected near St. Margarethen, Carinthia, Austria (46° 52’ 56.356”N, 14° 45’ 25.942’E). The soil is referred to as “Forst99” and was used in earlier studies (Lombi et al., 2000; Wenzel et al., 2002; Fitz et al., 2003). A compilation of physicochemical soil parameters and characterization methods is provided in Table S1. Forst99 is characterized by a pH_CaCl2_ of 7.21, a CaCO_3_ content of 135 gkg^-1^, and a high pseudo-total (aqua *regia* extractable) As fraction of 2080 mg kg^-1^ ± 18.1 mg kg^-1^. The high As mass fraction in Forst99 originates from the As-rich parent material containing arseniosiderite (Ca_2_Fe_3_(AsO_4_)_3_O_2_·3(H_2_O)), which weathers to secondary Fe^III^ (oxy)hydroxide and releases As^V^ during soil formation (Lombi et al., 2000). Prior to filling into the experimental growth containers (rhizotrons and rhizoboxes), the Forst99 soil was air-dried, sieved (≤ 2 mm), moistened to 280 g kg^-1^ water content corresponding to 40% water holding capacity (WHC), and incubated in the dark at 20 °C for 40 days.

### 2.2 Plant materials and growth conditions

Sporophytes of *P. vittata* (PV) and *P. quadriaurita* (var. tricolor; PQ) were purchased from ARC Ferns LLC (Apopka, FL, USA) and Der Palmenmann® (Castrop Rauxel, Germany), respectively. Ferns were pre-cultivated in a peat, perlite and compost-containing potting mix (pH 5.0-6.5) for 4-5 weeks. After the ferns had developed ~6 fronds, healthy PV and PQ specimen of similar size were selected and planted into pre-incubated Forst99 soil in the different experimental setups. The plants were grown in a controlled greenhouse environment at day/ night temperatures of 22 °C/17 °C (14 h/10 h), a minimum light intensity of 300 μmol m^-2^ s^-1^, and 60-70% relative humidity.

### 2.3 Solute imaging in the rhizosphere

#### 2.3.1 Reagents and analytical procedures

Unless stated otherwise, chemical reagents were of analytical grade and purchased from Alfa Aesar (Ward Hill, MA, USA), Merck (Darmstadt, Germany), Sigma-Aldrich (St. Louis, MO, USA) or VWR (Radnor, PA, USA). Glassware and plastics used for DGT gel preparation and deployment were acid-cleaned using 5% (*w*/*w*) HNO_3_ and rinsed three times with laboratory water type 1 (≤0.055 μS cm^-1^; TKAGenPure, Thermo Electron LED GmbH, Niederelbert, Germany). DGT gel solutions and reagents were prepared with laboratory water type 1. DGT coating and handling was performed in a biological class II laminar flow bench (Clean Air, EuroFlow EF/S, Telstar Laboratory Equipment B.V., Woerden, The Netherlands). All mass spectrometric analyses were conducted in a cleanroom (ISOclass 8 according to ISO14644-1).

#### 2.3.2 Rhizotron setup

**Fig. 1:**
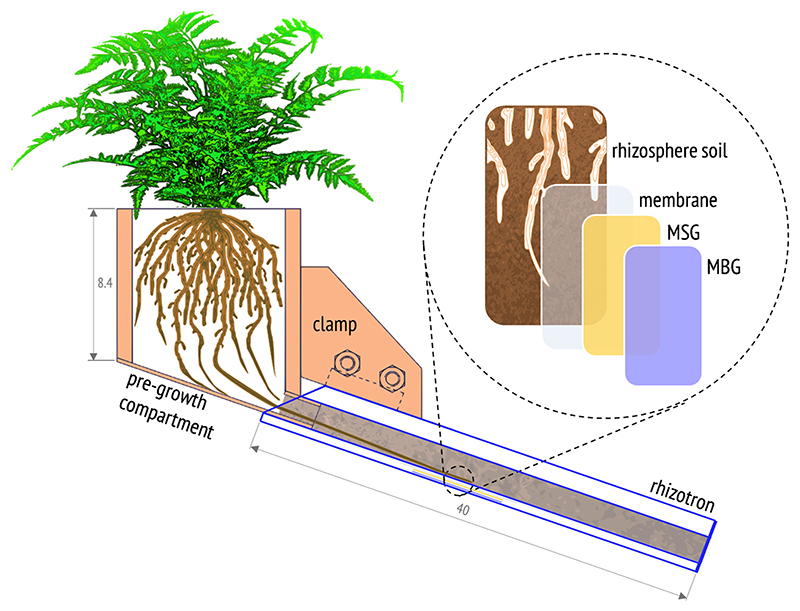
Scheme of the rhizotron experimental design (not to scale). The illustration shows the rhizotron assembly and the dual-layer MSG/MBG DGT probe setup for 2D solute imaging of As^III^, As^V^, P, Mn and Fe in the rhizosphere of hyperaccumulator ferns. Values represent cm.

Twelve acrylic rhizotrons (inner dimensions (i.d.) of *H* × *W* × *D* = 40 cm × 10 cm × 1.5 cm) with removable front plates and 14 irrigation drillings on the backside were equipped with tightly fitting pregrowth compartments (i.d. 8 cm × 8 cm × 8.4 cm; Technisches Büro für Bodenkultur, Auersthal, Austria) (Fig. 1). The rhizotron and pregrowth compartments were filled with pre-incubated Forst99 soil to a dry bulk density of 0.95 gcm^-3^ ± 0.05 gcm^-3^ (mean ± standard deviation (SD), *n* = 24). This ensured a homogeneous soil profile with adequate porosity for root growth. Before the removable front plate was attached, the soil was covered with a 50 μm-thin PTFE-foil (Haberkorn, Wolfurt, Austria) overlain by a PE-foil to allow opening the rhizotron upon sampling with minimal soil and root disturbance. Six precultivated fern specimen per species were transferred into soil-filled pre-growth compartments. Fern roots reached the bottom opening of the pre-growth compartments 28 days after planting (DAP) in Forst99 soil and were connected to the rhizotrons at this time. The assembly was inclined at ~ 25° to ensure root development along the removable front plate. For irrigation, rhizotrons were weighed twice per week and deionized water (≤0.2 μS cm^-1^, Millipore Elix 3, MilliporeSigma, Burlington, MA, USA) was added to maintain Forst99 at ~ 60% WHC during growth.

#### 2.3.3 Diffusive gradients in thin films (DGT) solute imaging

For simultaneous solute imaging of As^III^, As^V^, P, Mn and Fe in the rhizosphere, we used a novel approach, combining previously published DGT-mass spectrometric procedures (Bennett et al., 2011; Kreuzeder et al., 2013). We applied As^III^-specific DGT gels (MSG) and anion and cation mixed binding DGT gels (MBG) as a dual-layer gel sandwich in order to separate inorganic As species (i.e. As^III^ and As^V^) on MSG and MBG *in situ* during solute sampling. In this setup (Fig. 1), the MSG was placed onto the MBG and faced towards the soil-root interface to force As^III^ binding to MSG only. The thiol (-SH) functional groups of the 3-mercaptopropyl-functionalized silica gel resin in the MSG have been shown to selectively bind As^III^ but not As^V^ or organic As species (Howard et al., 1987; Bennett et al., 2011) and thus enable targeted sampling of labile As^III^ in the rhizosphere porewater. The original MSG gel fabrication procedure of Bennett et al. (2011) was modified to produce ~ 100 μm-thin DGT gels in a polyurethane gel matrix where the As^III^-selective binding resin was incorporated homogenously. Details on MSG fabrication can be found in the Supporting Information. The MBG was produced according to Kreuzeder et al. (2013) and consisted of the same gel matrix and had the same thickness as the MSG, but contained a mixture of suspended particulate reagent-iminodiacetate (SPR-IDA; CETAC Technologies, NE, USA) and zirconiumhydroxide precipitate (ZrOH), binding both labile trace metals (Mn and Fe) and oxyanions (As^III^, As^V^ and P).

Dual-layer DGT probes were deployed for 24 h on selected regions of interest alongside individual PV and PQ roots grown in the rhizotron setup. The soil water content was increased from ~ 60% to ~ 80% WHC 24 h before solute sampling to ensure optimal diffusion properties while avoiding anoxic soil conditions. A 10 μm-thin polycarbonate membrane (0.2 μm pore size; Nuclepore, Whatman, Maidstone, UK) served as diffusion layer between soil and dual-layer DGT. The gels were applied between 58 and 66 DAP for PV, and 65 and 72 DAP for PQ. The later gel application was due to slower growth of PQ roots.

After dual-layer DGT sampling, an excimer-based 193 nm laser ablation (LA) system (NWR193, ESI, NWR Division, Portland, OR, USA) coupled to a quadrupole ICP-MS (NexION 350D, Perkin Elmer, Waltham, MA, USA) was operated in line-scan mode for the spatially-referenced multi-element analysis of As, P, Mn and Fe on the dried MSG and MBG surfaces. Operating parameters of the LA-ICP-MS analysis (Table S2) were set to achieve a final pixel (i.e. datapoint) size in the solute images of 113 μm × 400 μm. The acquired data for each *m/z* was gas blank corrected, normalized using ^13^C as internal standard (Kreuzeder et al., 2013), and quantified using external calibration with both matrix-matched As^III^ (MSG), and As^V^, P, Mn and Fe (MBG) calibration standards. Refer to the Supporting Information for details on the DGT LA-ICP-MS calibration standard preparation and method detection limits (MDLs; Table S3). Data processing was conducted using a custom-written VBA macro in Excel, Version 2016 (Microsoft, Redmond, WA, USA). After calibration of the normalized signal intensities to solute loadings per gel area, μg cm^-2^, DGT-labile metal(loid) flux equivalents, f_DGT_, were calculated (Kreuzeder et al., 2013).

#### 2.3.4 Planar optode O_2_ imaging

1maging of oxygen (O_2_) distributions in rooted soil of PV and PQ was accomplished by color ratiometric planar optode imaging according to Larsen et al. (2011). The O_2_-optodes used in our study were based on a O_2_-quenchable platinum(II)octaethylpor-phyrin (PtOEP) fluorophore (Borisov, 2018). O_2_-optodes were deployed in parallel, at different regions of interest, to the dual-layer DGT probes in the corresponding PV and PQ replicates. After ~ 1 h incubation with deployed O_2_-optodes in the growth room, the rhizotrons were transferred to a dark room and positioned on a horizontal glass plate perpendicular to a digital single lens reflex camera (Canon EOS 1000D, Canon Inc., Tokyo, Japan) equipped with a macro lens (SIGMA 50 mm F2.8 DG MACRO, Sigma Corporation, Kanagawa, Japan). Optical filters and excitation LEDs were used as described in Larsen et al. (2011). The camera settings were ISO: 100, Av: f5.6, and Tv: 1/30 s. Details on O_2_-optode fabrication and calibration can be found in the Supporting Information.

#### 2.3.5 Image generation and data evaluation

The image processing software Fiji ImageJ 1.52p (National Institute of Health, Bethesda, MD, USA) was used for 2D image plotting, calculation, and evaluation. A Y/X scale factor of 3.53 without pixel interpolation was applied to recalculate pixel dimensions in the DGT images before their lossless export as TIFF files and figure arrangement in InDesign CS6 (Adobe, San Jose, CA, USA). For image evaluation, solute images and corresponding photographs were scale-matched and evaluated first visually for metal(loid) flux or O_2_ gradients between rhizosphere and bulk soil. 1f gradients to the corresponding bulk soil were visible, average solute fluxes and/or O_2_ values were calculated for the respective rhizosphere areas and compared to the average fluxes in the bulk soil. For solute flux quantifications in the rhizosphere, DGT images were first rotated to vertically align the root axis in the image center, before average solute fluxes were extracted from rectangular areas in the rhizosphere. These areas were defined as areas of increased solute flux, if their average fluxes were substantially higher (i.e. > average of corresponding adjacent area + 3 × SD) compared to the adjacent areas. Decreased, moderately decreased, and slightly decreased solute fluxes were assigned if the average fluxes in the rhizosphere were substantially lower (i.e. < average of corresponding bulk soil - 3 × SD), moderately lower (i.e. < average of corresponding bulk soil - 2 × SD), or slightly lower (i.e. < average of corresponding bulk soil - 1 × SD) compared to the bulk soil fluxes. To determine the spatial extent of these areas, the solute images were imported in Photoshop CS3 (Adobe, San Jose, CA, USA) and the maximum width of the areas of increased and decreased fluxes was measured using the ruler tool following image scaling. This value was subtracted from the average root diameter of the corresponding root surfaces (0.51 mm ± 0.04 mm; *n* = 7) and divided by two to obtain the distance of solute flux features from the root surface into each side of the rhizosphere soil (termed ‘beyond the root surface’ throughout this paper). Solute flux and distance values in text are reported as means ± SD.

### 2.4 Phytic acid analysis in root exudates of soil-grown P. vittata

#### 2.4.1 Rhizobox setup

Twelve rhizoboxes (Wenzel et al., 2001) were filled with Forst99 soil to achieve a consistent bulk density of ~1.2gcm^-3^. The rhizoboxes were equipped with soil-filled pre-growth compartments in which two pre-cultivated fern specimen per species were planted each. During growth, the soil water content in the rhizobox assembly was gravimetrically kept constant at ~ 60% WHC.

#### 2.4.2 Exudate sampling

Fern root exudates were sampled using a root exudate collection system (REC) in the rhizobox setup according to Oburger et al. (2013). Over the course of the three-month experimental growth period, roots of PQ did not develop the dense root mat required for this approach. Therefore, PQ was excluded from the exudate analysis. Sampling of PV root exudates was conducted 85 DAP over one day/night cycle (24 h) at four different sub-units on each PV root mat. All exudates were collected in a matrix of 10 mg L^-1^ Micropur Classic (Katadyn Products Inc., Kemptthal, Switzerland) to prevent microbial exudate decomposition. At the end of the sampling period, the four exudate-containing solutions from each sub-unit were pooled for each rhizobox replicate (n = 6). Recovery of exudates by the REC (75% ± 5%; *n* = 3) on Forst99 soil was determined as detailed in Oburger et al. (2013).

#### 2.4.3 Phytic acid analysis

Phytic acid in PV root exudates was analyzed by high-performance ion chromatography (1CS-3000DP, Thermo Fisher Scientific, Waltham, MA, USA) combined with ICP-MS (Elan 6100 DRC II, PerkinElmer, Woodbridge, Ontario, Canada) according to Rugova et al. (2014).

### 2.5 Fern biomass analysis

Ferns grown in the rhizotron experiment were immediately harvested after DGT and O_2_-optode application (52-67 DAP for PV and 66-73 DAP for PQ). Ferns were separated into roots (including the rhizome) and fronds. All biomass samples were thoroughly washed with deionized water (≤0.2 μS cm^-1^) in an ultrasonic bath, rinsed several times with laboratory water type 1 (≤0.055 μS cm^-1^) gently blotted, dried at 65 °C for 72 h, and the weights were recorded. Finely ground and homogenized subsamples (200 mg) of the plant material were digested in a 5:1 (*v*/*v*) mixture of HNO_3_ (65%, *w*/*w*; EMPARTA, ACS, Merck), H_2_O_2_ (30%, *w/w*; TraceSELECT Ultra, Fluka, Sigma-Aldrich) and a drop of iso-Octanol in open Pyrex tubes using an electrical heating block system (DK Heating Digester, Velp Scientifica, Usmate Velate, Italy). For quality control, three blanks and certified plant reference material (Oriental Basma Tobacco Leaves, InCT-OBTL-5, Institute of Nuclear Chemistry and Technology, Warsaw, Poland) were included in each digestion run. The digests were filtered (0.45 μm; Munktell 14/N, Munktell, Bärenstein, Germany) and diluted with laboratory water type 1 to reach a 2% (*w*/*w*) HNO_3_ matrix. Elemental concentrations in the fern digests were measured using ICP-OES (Optima 8300, Perkin Elmer, Waltham, USA) and ICP-MS (Elan 9000 DRCe, Perkin Elmer, Waltham, USA) with internal standardization (^115^In) and external multi-point calibration (ICP multi-element standard VI, Certipur®, Merck).

### 2.6 Statistical analysis

One-way analysis of variances (ANOVAs) were calculated for testing significant differences of As and P mass fractions between PV and PQ root and frond biomasses at *p* ≤ 0.05. Statistical analysis was conducted in IBM SPSS Version 23 (IBM Corporation, Armonk, NY, USA).

## 3 Results

### 3.1 Fern biomass production and elemental composition

**Fig. 2:**
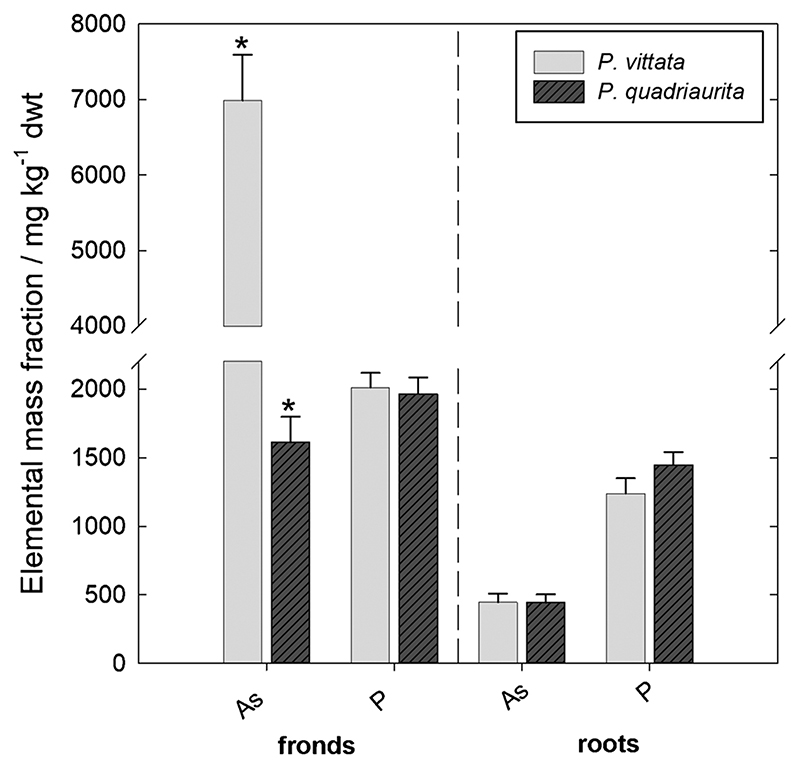
Average As and P mass fractions in frond and root tissue dry-weights (dwt) of PV (harvested between 52 and 67 DAP; *n* = 5) and PQ (harvested between 66 and 73 DAP; *n* = 4) after growth in rhizotrons filled with Forst99 soil. Asterisks indicate significant differences (*p* ≤ 0.05). Error bars display the standard error of the mean.

After ~ 2 months of growth in As-rich Forst99 soil, dry-weight biomass production was not different for the two fern species *(p* > 0.05), with 3.31 g **±** 0.70 g of fronds and 1.96 g **±** 0.66 g of roots for PV (mean ± SD, *n* = 5), and 3.12 g ± 0.81 g of fronds and 2.04 g ± 0.54 g of roots for PQ (mean ± SD, *n* = 4). However, PV showed isolated necrotic areas at the pinnae margins of a few senescing fronds, whereas PQ did not develop visual toxicity symptoms. Fern tissue As and P mass fractions are presented in Fig. 2. While As mass fractions in fronds were 4-fold higher in PV than in PQ (p ≤ 0.05), As mass fractions in roots of the fern species were virtually identical (p > 0.05). Consequently, PV showed a 4-fold higher As frond-to-root ratio (i.e. translocation factor; 14.1; *n* = 5) as compared to PQ (3.71; *n* = 4). Frond and root P mass fractions were not different between PV and PQ (*p* > 0.05).

### 3.2 Solute flux distribution of As^III^, As^V^, P, Mn and Fe

**Fig. 3:**
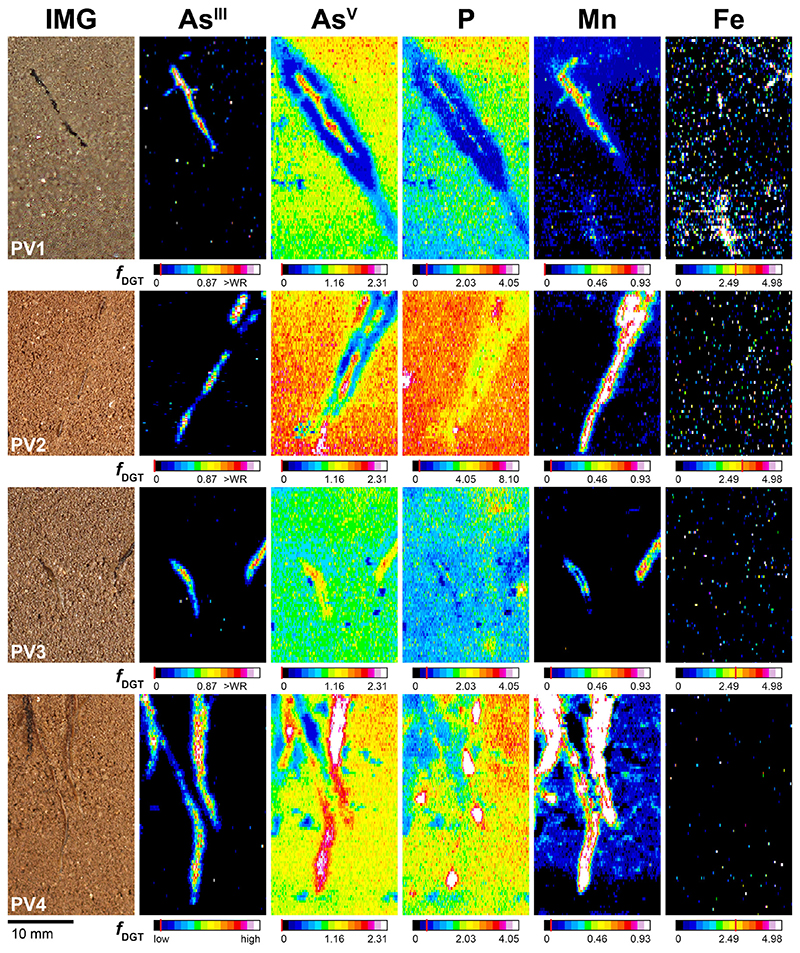
Solute images of labile As™, As^V^, P, Mn and Fe in the rhizosphere of PV after 24 h of dual-layer DGT application, 64 (PV1), 58 (PV2, PV3) and 66 (PV4) DAP in rhizotrons filled with Forst99 soil. Solutes are shown as metal(loid) fluxes,f_DGT_, in pg cm^−2^ s^−1^. For PV4, the As^III^ map shows qualitative data only, because the corresponding MSG gel was accidentally analyzed upside down, i.e. at the gel surface area which faced the MBG gel. Note that As^III^ images are slightly rotated and offset compared to As^V^, P, Mn and Fe images, as the MSG was not perfectly overlying the MBG during dual-layer DGT sampling. Analyte MDLs are indicated by the red line in the corresponding calibration bars. WR denotes the upper limit of the calibration working range. (For interpretation of the references to colour in this figure legend, the reader is referred to the web version of this article).

**Fig. 4:**
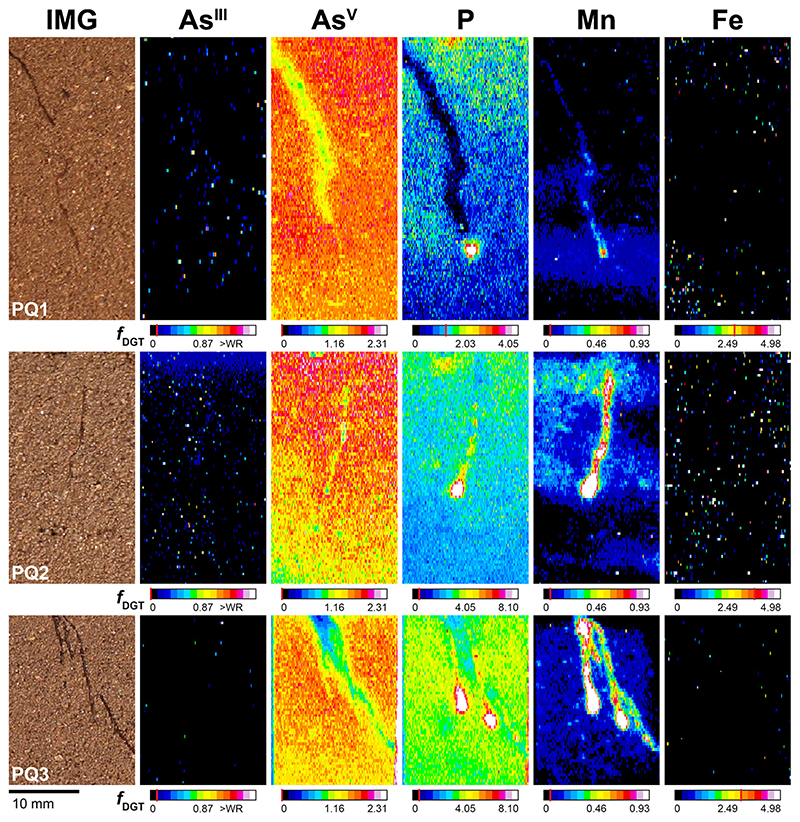
Solute images of labile As^III^, As^V^, P, Mn and Fe in the rhizosphere of PQ after 24 h of dual-layer DGT application, 70 (PQ1, PQ2) and 72 (PQ3) DAP in rhizotrons filled with Forst99 soil. Solutes are shown as metal(loid) fluxes, fDGT, in pg cm^-2^ s^-1^. Note that As^III^ images are slightly rotated and offset compared to As, P, Mn and Fe images, as the MSG was not perfectly overlying the MBG during dual-layer DGT sampling. Analyte MDLs are indicated by the red line in the corresponding calibration bars. WR denotes the upper limit of the calibration working range. (For interpretation of the references to colour in this figure legend, the reader is referred to the web version of this article).

The 2D As^III^, As^V^, P, Mn and Fe solute flux distribution in the rhizosphere and bulk soil of four PV (PV1 - 4) and three PQ (PQ1- 3) replicates are presented in Figs. 3 and 4. Areas from which solute flux values were extracted are shown in Figs. S1 (PV) and S2 (PQ). For enhanced visualization of the fine-scale solute flux patterns in the rhizosphere of PV, an exemplary profile plot across the soil-root interface of one PV replicate (PV1) is provided in Fig. S3. The highest total As^III^ gel loading at any single MSG location (i.e. over the whole data range) was 2.91 nmol cm^-2^ and thus well below the reported MSG As^III^ binding capacity of 329 nmol cm^-2^ (Bennett et al., 2011). For MBG, anion and cation gel loadings were generally also well below the respective anion (230 nmol cm^-2^) and cation (310 nmol cm^-2^) MBG binding capacities (Kreuzeder et al., 2013), except for some isolated, almost single-pixel areas where Fe exceeded the MBG capacity up to 3-fold (Fig. 4, PQ2). This ensured zero sink conditions during deployment of the dual-layer DGT probes.

#### 3.2.1 Solute flux in bulk soil

The average background flux of As^III^, As^V^, P, Mn and Fe in bulk soil of all PV and PQ replicates is presented in Table 1. Labile As^III^ was consistently below the As^III^ MDL (Tab. S3). In contrast, labile As^V^ showed a homogeneous distribution with an average As^V^ flux of 1.37 pg cm^-2^ s^-1^ ± 0.26 pg cm^-2^ s^-1^ across all PV and PQ bulk soil areas. Compared to As^V^, the bulk distribution of labile P was more heterogeneous within and between PV and PQ replicates. Labile Mn and Fe were consistently below or close to the Mn MDL and Fe MDL, respectively (Tab. S3).

#### 3.2.2 Solute flux in the rhizosphere of P. vittata

Distinct As^III^ flux increases were mapped in the rhizosphere of all PV replicates (Fig. 3). The As^III^ increases occurred as elongated areas closely associated with individual PV root axes, extending on average up to 0.5 mm ±0.1 mm (n = 4) beyond the root surface (Fig. S3). At locations where the root surface was covered by a thin soil layer, and therefore not in direct contact with the dual-layer DGT, As^III^ fluxes diminished (Fig. 3, PV1 and PV2). Flux maxima of As^III^ up to 2.52 pg cm^- 2^ s^-1^ were measured at the very root surface of mature root segments (Fig. 3, PV2). Only minor relative increase was found at apical root segments (Fig. 3, PV3 and PV4). For example, at the root tip of PV3, the average As^III^ flux was 0.39pg cm^-2^ s^-1^ ± 0.13pg cm^-2^ s^-1^, corresponding to a 58% As^III^ flux decrease as compared to the subapical region of the same root (Fig. 3, PV3).

Mapping of As^V^ revealed a highly heterogeneous As^V^ distribution in the vicinity of PV roots (Fig. 3). We found elongated areas of increased As^V^, which were consistently co-localized with the areas of increased As^III^ but extended generally a little farther (0.6 mm ± 0.2 mm) beyond the root surface (Fig. S3). Flux maxima of As^V^ up to 3.85 pg cm^-2^ s^-1^ were determined in PV4, at the location where the surfaces of two adjoining PV roots were in direct contact with the dual-layer DGT (Fig. 3, PV4). The areas of increased As^III^/As^V^ flux were consistently localized within areas of decreased As^V^, where As^V^ fluxes were depleted down to minimum values of 0.27 pg cm^-2^ s^−1^ (Fig. 3, PV1). The distinct zones of As^V^ depletion in PV1 and PV2 extended up to 2.2 mm (Fig. S3) and 1.5 mm beyond the root surface and accounted for a 67% and 61% As^V^ flux decrease, respectively, compared to the average As^V^ flux in the respective bulk soils (Fig. 3, PV1 and PV2). Slightly decreased As^V^ fluxes were also observed alongside apical and sub-apical root segments (Fig. 3, PV3 and PV4).

Co-localized with the areas of increased As^III^/As^V^, elongated areas of increased P and Mn were mapped in the PV rhizosphere (Fig. 3). The increased P fluxes were localized at the immediate root surface (Fig. 3, PV1, PV2 and PV4), whereas the increased Mn fluxes extended 0.8 mm ± 0.2 mm beyond the root surface and thus ~0.2 mm beyond the areas of increased As^III^/As^V^ (Fig. S3). At PV4 root tips and apical root segments, drop-shaped areas with substantially increased P and Mn flux maxima up to 28.3 pg cm^-2^ s^-1^ and 10.1 pg cm^-2^ s^-1^, respectively, were observed (Fig. 3, PV4). The areas of increased P were found within areas of moderately (Fig. 3, PV1, PV2 and PV4) and slightly (Fig. 3, PV3) decreased P flux in the rhizosphere. Congruent to As^V^, P depletion extended up to 2.2 mm beyond the root surface, lowering P fluxes down to 60% as compared to the respective bulk soil (Figs. 3 and S3, PV1). Labile Fe in the rhizosphere remained consistently below the Fe MDL, with no relative changes in rhizosphere compared to bulk soil (Fig. 3).

#### 3.2.3 Solute flux in the rhizosphere of P. quadriaurita

In the rhizosphere of PQ, As^III^ remained consistently below the As^III^ MDL, no areas of increased As^III^ or As^V^ fluxes were mapped (Fig. 4). Distinct depletion of As^V^ alongside individual PQ roots was observed in two out of three PQ replicates, with As^V^ flux minima of 0.38 pg cm^-2^ s^-1^ at the location where three adjoining PQ roots were in direct contact with the dual-layer DGT (Fig. 4, PQ3). In one PQ replicate areas of decreased As^V^ in the rhizosphere were observed only visually and not according to the defined classification criteria (Fig. 4, PQ2). Depletion of As^V^ fluxes in PQ1 and PQ3 extended up to 0.7 mm and 0.3 mm beyond the root surface, accounting for a 24% and 37% As^V^ flux decrease, respectively, as compared to the respective bulk soils (Fig. 4, PQ1 and PQ3). Thus, As^V^ depletion in the rhizosphere was less pronounced for PQ as compared to PV.

For P and Mn, co-localized areas of highly increased fluxes around root tips were mapped in all PQ replicates, reaching P and Mn flux maxima of 78.9 pg cm^-2^ s^-1^ and 9.07 pg cm^-2^ s^-1^, respectively (Fig. 4, PQ2). With increasing distance to the root tip, P and Mn fluxes generally decreased. Two out of three PQ replicates showed, however, distinct areas of elongated Mn increase at mature root parts, extending up to 0.7 mm beyond the root surface (Fig. 4, PQ2 and PQ3). For P, solubilization hotspots at root tips extended towards moderately/ slightly decreased P fluxes around mature PQ root segments (Fig. 4, PQ1 and PQ3). As for PV, labile Fe fluxes in the rhizosphere of PQ remained below the Fe MDL (Fig. 4).

### 3.3 Oxygen (O_2_) distribution in rooted soil ofP. vittata and P. quadriaurita

**Fig. 5:**
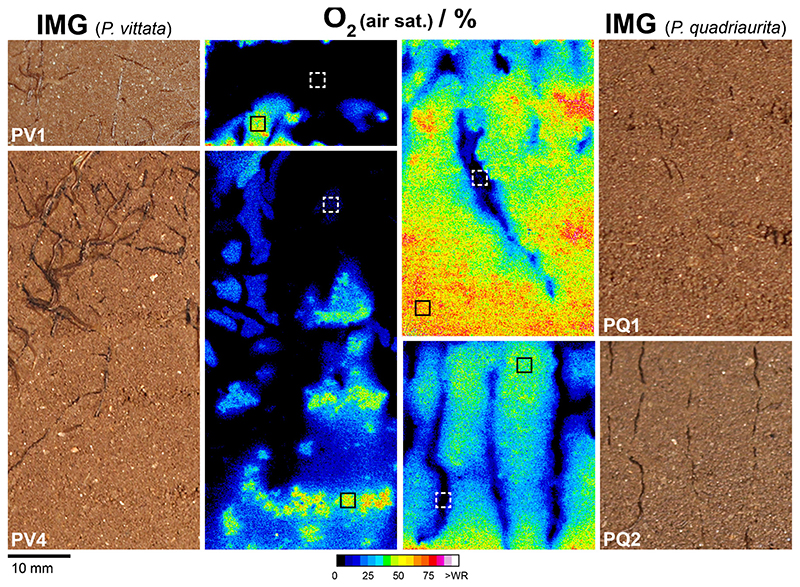
Distribution of O_2_ levels (percent air saturation), mapped by planar O_2_-optodes, in rooted soil of PV and PQ, 64 (PV1), 66 (PV4) and 70 (PQ1 and PQ2) DAP in rhizotrons filled with Forst99 soil. Framed locations in the O_2_ images depict the areas (A = 4.24 mm^2^) where average O_2_ levels in bulk (solid black rectangles) and rooted soil (dashed white rectangles) were extracted *(n* = 400). (For interpretation of the references to colour in this figure legend, the reader is referred to the web version of this article).

Planar O_2_-optode measurements showed a rapid and extensive depletion of O_2_ in the rhizosphere of PV and PQ (Fig. 5). The O_2_ air saturation measured in selected bulk soil and rhizosphere areas (*A* = 4.24 mm^2^, *n* = 400) revealed an average decrease of O_2_ from up to 48.9% ± 0.8% (*n* = 2) in bulk soil to 2.51% ± 3.51% (*n* = 2) in the densely rooted soil of PV (Fig. 5, PV1 and PV4). For PQ, O_2_ air saturation was decreased from 51.6% ± 17.5% (*n* = 2) in bulk soil to 4.62% ± 0.37% (n = 2) alongside roots (Fig. 5, PQ1 and PQ2). Thus, PV and PQ showed similar patterns of O_2_ depletion in terms of reduced O_2_ availability. For PQ, however, the spatial extent of the lowered O_2_ availability was more confined to the immediate root surroundings (Fig. 5). Note that multiple roots located just behind the soil surface might have contributed to the substantial O_2_ decrease.

### 3.4 Phytic acid in root exudates of P. vittata

In all investigated PV rhizobox replicates (*n* = 6) the content of phytic acid in root exudate samples was below the method detection limit (MDL) of 4.8 nmol L^-1^.

## 4 Discussion

### 4.1 Arsenic accumulation in P. vittata and P. quadriaurita

Biomass analysis revealed significant differences in As accumulation between PV and PQ (Fig. 2). For PV, strong As root uptake, translocation and frond accumulation confirmed As hyperaccumulation characteristics (Ma et al., 2001; Fitz et al., 2003). In contrast, PQ was evidently less efficient in As uptake and translocation than PV, as shown by the significantly lower As translocation factor and the significantly lower As mass fractions in fronds compared to soil (i.e. bioconcentration factor). Yet, both PV and PQ showed almost identical total root As mass fractions with 445 mg kg^− 1^ ± 141 mg kg^− 1^ for PV (mean ± SD, *n* = 5) and 444 mg kg^− 1^ ± 119 mg kg^− 1^ for PQ (mean ± SD, *n* = 4). Moreover, molar P/As ratios in root tissues were not significantly different with 7.30 ± 2.82 for PV (mean ± SD, *n* = 5) and 8.21 ± 1.66 for PQ (mean ± SD, *n* = 4). This suggests that both fern species tightly control the root As and P levels to avoid interferences of As with plant P metabolism. In PV, however, the strong As translocation from roots to fronds resulted in necrotic effects at pinnae margins of senescing fronds and low frond molar P/As ratios of 0.71 ± 0.13 (mean ± SD, *n*= 5). This indicates moderate As toxicity in PV due to As stress (Lombi et al., 2002; Tu and Ma, 2003, 2005; Gonzaga et al., 2009; Han et al., 2019), inducing internal As detoxification to sustain P nutrition at adequate levels for plant growth. PQ did not show necrosis at fronds and the molar P/As ratios of 3.01 ± 0.37 (mean ± SD, *n* = 4) in fronds were in line with previous work where PQ was grown in As-spiked soil (100 mg kg^-1^) without suffering from As toxicity (Srivastava et al., 2006). Contrary to the moderately elevated soil As levels in the study of Srivastava et al. (2006), where both As translocation and bioconcentration factors were clearly indicative for As hyperaccumulation by PQ, the high soil As levels (2080 mg kg^-1^) in the present study resulted in As accumulation typical for As-tolerant plant species (Meharg and Hartley-Whitaker, 2002; Fitz and Wenzel, 2006). Thus, PQ appears to use an adaptive strategy to cope with As toxicity, where it acts as an hyperaccumulator if As exposure is moderate (Srivastava et al., 2006), and as an As-tolerant ecotype if As exposure is high (the present study). To the best of our knowledge, this mechanism has not been observed before in other plant species and thus warrants further investigations in targeted dose-response experiments.

### 4.2 Root-induced modulation of O_2_ availability at the soil-root interface

Planar O_2_-optode measurements visualized extensive O_2_ depletion in the immediate root vicinity of both ferns (Fig. 5), suggesting partly anoxic conditions and thus a locally lowered redox potential in the rhizosphere as observed earlier (Fitz et al., 2003). Although the planar optode images are not providing direct mechanistic insight into the origin of the O_2_ depletion (Blossfeld, 2013), it has been shown before that enhanced root and microbial respiration in the carbon-rich root zone of terrestrial plants can cause the formation of transient O_2_ gradients between rhizosphere and bulk soil (Rudolph-Mohr et al., 2012; Hoefer et al., 2015). Upon rapid consumption of the energetically most favorable terminal electron acceptor (TEA), O_2_, the reduction of less favorable TEAs, NO_3_^-^ and Mn^III/IV^, proceeds quickly (McBride, 1994), resulting in rapid Mn^II^ release into the rhizosphere solution as shown by the areas of elongated Mn flux increase alongside PV and PQ roots.

Strong O_2_ depletion in the rhizosphere can cause reducing conditions resulting in direct abiotic As^V^ reduction to As^III^ (Masscheleyn et al., 1991). However, as the areas of increased As^III^ flux were confined to the immediate root surface of PV only, while the zones of O_2_ depletion covered extensive areas across the soil-root interface of both PV and PQ, the lowered O_2_ availability appears to be insufficient to cause reducing conditions which could result in abiotic As^V^ reduction to As^III^. This is corroborated by the consistent lack of labile Fe in the O_2_-depleted rhizospheres of both fern species. The As^V^/As^III^ and Fe^III^/Fe^II^ redox couples share very similar transition thresholds in soil (between 0mV and -100 mV at pH 7; Borch et al., 2010). Thus, the absence of changes in labile Fe between rhizosphere and bulk soil confirms that the redox potential remained sufficiently high for Fe and hence As to remain in their oxidized forms. This was initially surprising when compared to Fitz et al. (2003), who found 3-fold higher soil porewater Fe concentrations in PV rhizosphere than in bulk soil using a rhizobox setup and the same experimental soil. However, accumulation of labile Fe would imply acidic pH < 6.5 and prolonged anoxic conditions if labile Fe is released during siderite dissolution (Renard et al., 2017) or dissimilatory Fe^III^ (oxyhydr)oxide reduction by microorganisms (Lovley et al., 2004). Siderite dissolution primarily releases insoluble FeCO_3_(aq) at pH > 6.5 or Fe(CO_3_)_2_^2-^ at pH > 8.5 and is regarded a slow process (Bruno et al., 1992). Labile Mn^II^ is kinetically relatively stable at neutral pH even in the presence of O_2_ (Tebo et al., 2005), and oxidation to Mn^III/IV^ is much slower compared to oxidation of Fe^II^ to Fe^III^ phases (Morgan, 2000). Therefore, the apparent discrepancy between our results and the findings of Fitz et al. (2003) may be explained by the different experimental approaches, with reductive Fe solubilization proceeding only under the influence of a dense root mat, but not along the individual roots investigated in the present study.

### 4.3 Spatial patterns of arsenic cycling in the rhizosphere

Simultaneous, sub-mm scale solute imaging of labile As^III^ and As^V^ fluxes by dual-layer DGT LA-ICP-MS visualized differential patterns of As redox transformations showing different mechanisms of As cycling at the soil-root interface of individual PV and PQ roots. Consistent with more efficient As accumulation by PV, we found 2-fold higher depletion of labile soil As^V^ in the rhizosphere of PV (Figs. 3 and 4), indicating an overall higher As^V^ root uptake efficiency of PV compared to PQ. The observed As^V^ depletion in the PV rhizosphere is in line with previous evidence from both hydroponic (Wang et al., 2002; Poynton et al., 2004) and soil-based (Fitz et al., 2003; Gonzaga et al., 2006) experiments and shows that individual PV roots act as a strong localized sink for labile As^V^ in As-rich soil, depleting labile As^V^ fractions up to ~ 2 mm from the root surface into the adjacent soil. At the immediate soil-root interface (i.e. the rhizoplane), the local As speciation and associated P and Mn solute flux patterns differed largely between PV and PQ. Only PV showed steep As^III^ gradients which were co-localized with increased As^V^, P and Mn fluxes in the center of the As^V^ depletion zones, whereas indications for root-induced As redox transformations were consistently missing in the rhizosphere of PQ (Figs. 3 and 4).

The localized As^III^ flux increase at the PV rhizoplane is consistent with enhanced As^V^ reductase activity in root tissues (Liu et al., 2009; Cesaro et al., 2015) and As^III^ root efflux (Chen et al., 2016; Han et al., 2016) by PV when grown in As^V^-rich growth media, and shows that individual PV roots can change As speciation in the rhizosphere (As^III^/ As^V^ ratio of 0.57) as compared to bulk soil (As^III^/As^V^ ratio of ≤0.04). Since As^III^ is, compared to As^V^, no chemical analogue to P, it does not interfere with plant P metabolism (Zhao et al., 2009). However, As^III^ is also toxic to plants due to reactions with thiol groups, disrupting enzymatic activity and protein synthesis (Meharg and Hartley-Whitaker, 2002). While non-accumulators typically cope with high cytoplasmic As^III^ concentrations via sequestration of As^III^-phytochelatin complexes in root vacuoles resulting in low As^III^ translocation to shoots (Zhao et al., 2009), PV shows only minor As^III^ complexation in roots and efficient As^III^ translocation to fronds (Su et al., 2008). Therefore, in line with previous results from hydroponic experiments (Su et al., 2008; Huang et al., 2011b; Chen et al., 2016; Han et al., 2016), a fraction of the As^III^ produced in PV root cells may be effluxed into the apoplasm and subsequently diffuse into the rhizosphere soil to lower the cellular As^III^ burden and thus avoid metabolic As^III^ stress. This As^III^ efflux mechanism may be essential for adaptive As detoxification by PV if As^V^ uptake and internal reduction to As^III^ exceeds As^III^ translocation rates to fronds (Wang et al., 2010).

Indeed, it has been proposed that As^III^ efflux may be a constitutive mechanism for As tolerance in plants (Gupta et al., 2011). However, the apparent lack of detectable As^III^ fluxes in the rhizosphere and hence As^III^ root efflux by PQ contrasts with this interpretation. The 2-fold lower As^V^ depletion in the rhizosphere of PQ compared to PV (Figs. 3 and 4), along with the 4-fold lower total As content in fern tissues (Fig. 2), indicate that As-tolerance by PQ involves an overall reduced As uptake compared to PV. Exclusion of As from root uptake is known from several non-hyperaccumulating As-tolerant plant species such as *Holcus lanatus, Deschampsia cespitosa* and *Cytisus striatus* (Meharg and Hartley-Whitaker, 2002; Bleeker et al., 2003). While PQ does not avoid As uptake, our data indicate that this species efficiently controls As uptake to avoid exceeding tolerable cytoplasmic As, and not effluxing As like PV.

The spatial patterns of As^III^ efflux in the PV rhizosphere were consistently co-localized with increased As^V^, P and Mn fluxes, pointing towards As^III^ re-oxidation in the rhizosphere through chemical oxidation of As^III^ to As^V^ by Mn^III/IV^ (oxyhydr)oxides (Oscarson et al., 1981, 1983; Li et al., 2010; Liu et al., 2012). Following oxidation of As^III^ on Mn^III/IV^ (oxyhydr)oxide surfaces, As^V^ and Mn^II^ are concomitantly released into solution, where they may readily bind to the available sorption sites, including the MBG in the dual-layer DGT probe. The As^V^ produced in this reaction can mobilize P via ligand exchange on mineral surfaces (Liu et al., 2001), which may explain the increased P fluxes at the immediate root surface (Fig. 3). We therefore hypothesize that the observed Mn solubilization was, apart from dissimilatory Mn reduction, linked to As cycling by PV, partially mediating As^III^ re-oxidation to As^V^. Redox cycling remained rapid as increased As^III^ fluxes were confined to the immediate root vicinity and As^V^ quantities were similar to those of As^III^ (Fig. S3), especially if the As^V^ background in the adjacent depletion zones was accounted for (As^III^/As^V^ ratio of 0.97). The fact that the areas of As^V^ and Mn co-localization showed almost an identical spatial extent as compared to those of As^III^ (Fig. S3) indicated only marginal diffusion of As^III^ from the rhizoplane into the soil and back towards the duallayer DGT probe during As^III^ efflux and re-oxidation. Thus, in line with previous evidence from soil incubation experiments (Ying et al., 2012; Ehlert et al., 2016), Mn^III/IV^ (oxyhydr)oxides may effectively retard diffusion of As^III^ from the rhizosphere into the bulk soil by promoting rapid As^III^ oxidation and subsequent As^V^ sorption to soil solid phases. However, rhizospheric oxidation of As^III^ to As^V^ may have also been mediated by microbes present at the root surface (Mathews et al., 2010).

Interestingly, the spatial pattern of As^III^ root efflux and re-oxidation in the PV rhizosphere consistently followed root development. Localized patterns of As^III^ efflux were observed in the zone of root maturation (Fig. 3, PV1 - 4), decreasing towards the root tips (Fig. 3, PV3 and PV4). This spatial zonation coincides with the reported root tissue distribution of aquaporins (Hachez et al., 2006), which are known to mediate bidirectional As^III^ transport along concentration gradients across PV root membranes (He et al., 2016). The small spatial extension of this diffusion-controlled mechanism may also explain why previous soil-based experiments could not detect As speciation and mobilization changes in the rhizosphere porewater (Fitz et al., 2003). While rapid diffusion of effluxed As^III^ in hydroponic experiments can lead to a complete reduction of As^V^ to As^III^ in the growth medium (Xu et al., 2007; Chen et al., 2016), retarded As^III^ diffusion in soil-based systems may confine As mobilization to the immediate (sub-mm) root vicinity (Kuppardt et al., 2010). The steep As^III^ /As^V^ gradients in the immediate vicinity of individual PV roots visualized in the solute images are in line with this assumption.

### 4.4 Phosphorus and manganese mobilization at root tips

Co-localization of highly increased P and Mn fluxes was observed at both PV and PQ root tips (Figs. 3 and 4). The P and Mn hotspots were not accompanied by increased As^III^ or As^V^ fluxes, revealing a surprising, spatially confined disconnect between P and Mn mobilization and As release. In earlier work, we observed similar P flux patterns with increased labile P at root tip regions of *Brassica napus*, *Fagopyrum esculentum*, *Lupinus albus* and *Triticum aevestivum* which were partly co-localized with increased Mn fluxes (Santner et al., 2012; Kreuzeder et al., 2018). These Mn features had been shown to be caused by localized rhizosphere acidification leading to Mn^III/IV^ (oxyhydr)oxide dissolution. Although *Pteris* species can also modify their rhizosphere pH (Gonzaga et al., 2006, 2009), rhizosphere acidification is unlikely to play an important role for Mn solubilization in the present study due to the high buffer capacity of the Forst99 soil for protons (Fitz et al., 2003). Besides, rhizodeposition of root border cells into the rhizosphere soil during root growth may have contributed to the co-localization of increased P and Mn fluxes at root tip areas. In ongoing work evidence for P rhizodeposition at root tips of several plant species, including *Zea mays*, *Helianthus annuus* and *Phaseolus vulgaris* has been found (Golestanifard and Santner et al., unpublished results). We thus suggest that rhizodeposition also contributes to the increased P and Mn fluxes around fern root tips observed in the present study.

### 4.5 Phytic acid exudation by P. vittata1

Despite previous reports on phytic acid exudation from PV roots in hydroponic culture (Tu et al., 2004b; Liu et al., 2016), we did not detect phytic acid in exudates sampled from soil-grown PV specimen. Interestingly, phytic acid exudation rates in the hydroponic study of Liu et al. (2016) increased with increasing As^V^ concentration in the external medium. If phytic acid would be released for mobilizing As from the soil for plant uptake, analogous to e.g. citrate release for mobilizing soil P by *Lupinus albus* (Neumann and Römheld, 1999), decreasing exudation rates with increasing external As concentrations would be expected, as the larger carbon and energy investment for As mobilization would be necessary in the case of low, but not high, external As availability. Since there are no other investigations of phytic acid release from PV roots available, further work is required to elucidate if phytic acid contributes to As hyperaccumulation by PV.

## 5 Conclusion

We visualized the sub-mm solute flux distribution of As^III^, As^V^, P, Mn, Fe and O_2_ along individual As hyperaccumulator roots grown for two months in soil geogenically enriched in As. Reproducible patterns in the rhizospheric As^III^/As^V^ distribution and total As accumulation in fern tissues revealed differential As uptake mechanisms and biotransformation pathways in PV and PQ. We conclude that the high As uptake, translocation and accumulation from As-rich soil in PV fronds induced the need for root As detoxification via As^V^ reduction and As^III^ efflux leading to As^III^ accumulation and re-oxidation to As^V^ in the rhizosphere porewater. This As cycling mechanism is accompanied by the reduction of O_2_ and Mn^III/IV^ (oxyhydr)oxides leading to decreased O_2_ availability and increased Mn solubilization along individual roots. In contrast to PV, we found no As^III^ efflux by PQ, but indication for PQ efficiently controlling As root uptake to tolerable levels. Interestingly, in the present study, As acquisition by PV was not associated with phytic acid release from roots, highlighting the need for further work to elucidate the role of phytic acid in As hyperaccumulation. Our study demonstrates that two closely-related As-accumulating fern species, *P. vittata* and *P. quadriaurita*, have distinct mechanisms for As uptake, and provides new evidence for the importance of species-specific plant adaptations in modulating As redox transformations and cycling in Asrich environments. Future work on PV and PQ should aim to determine the adaptive advantages of each mechanism.

## Supplementary Material

Supplementary material related to this article can be found, in the online version, at doi:https://doi.org/10.1016/j.envexpbot.2020.104122.

SI

## Figures and Tables

**Table 1:** Average As^III^;, As^V^, P, Mn and Fe solute fluxes (± SD, *n* = 340) in bulk soil areas (A = 14.8mm^2^) of PV and PQ replicates.

		foGT in bulk soil (pg cm ^-2^ s^-1^)				
Fern species		As^III^	As^V^	P	Mn	Fe
*Pteris vittata*	PV1	≤0.07^a^	1.03 ± 0.10	1.41 ± 0.23	0.06 ± 0.02	≤2.72^a^
	PV2	≤0.03^a^	1.46 ± 0.14	5.47 ± 0.39	≤0.08^a^	≤3.25^a^
	PV3	≤0.05^a^	0.97 ± 0.08	1.55 ± 0.27	≤0.04^a^	≤2.66^a^
	PV4	n.c.^b^	1.22 ± 0.08	2.22 ± 0.24	0.10 ± 0.06	≤2.66^a^
*Pteris quadriaurita*	PQ1	≤0.07^a^	1.55 ± 0.13	≤1.34^a^	0.06 ± 0.02	≤2.81^a^
	PQ2	≤0.05^a^	1.41 ± 0.19	2.37 ± 0.32	0.07 ± 0.20	≤3.65^a^
	PQ3	≤0.07^a^	1.44 ± 0.11	3.50 ± 0.28	≤0.08^a^	≤319^a^

a Method detection limit (MDL).

b Not calibrated.
