# Supplementary material for: Arsenic redox transformations and cycling in the rhizosphere of Pteris vittata and Pteris quadriaurita
Source: Environ Exp Bot. Author manuscript; Available in PMC 2021 Jun 7. (PMC7610922; doi:10.1016/j.envexpbot.2020.104122)
Supplement: SI [file EMS124812-supplement-SI.pdf]

SUPPORTING INFORMATION

Arsenic redox transformations and cycling in the rhizosphere of *Pteris vittata* and *Pteris quadriaurita*

Stefan Wagner[a,b,c,1], Christoph Hoefer[a,d,1], Markus Puschenreiter[a], Walter W. Wenzel[a], Eva Oburger[a], Stephan Hann[c], Brett Robinson[e], Ruben Kretzschmar[d], Jakob Santner[b,f,*]

[a]Department of Forest and Soil Sciences, Institute of Soil Research, Rhizosphere Ecology & Biogeochemistry Group, University of Natural Resources and Life Sciences, Vienna, Konrad-Lorenz-Strasse 24, 3430, Tulln, Austria.

[b]Department General, Analytical and Physical Chemistry, Chair of General and Analytical Chemistry, Montanuniversität Leoben, Franz-Josef-Strasse 18, 8700, Leoben, Austria.

[c]Department of Chemistry, Institute of Analytical Chemistry, University of Natural Resources and Life Sciences, Vienna, Muthgasse 18, 1190, Vienna, Austria.

[d]Department of Environmental Systems Science, Institute of Biogeochemistry and Pollutant Dynamics, Soil Chemistry Group, ETH Zürich, Universitätstrasse 16, CHN, 8092, Zürich, Switzerland.

[e]School of Physical and Chemical Sciences, University of Canterbury, 20 Kirkwood Ave, Ilam, Christchurch, 8041, New Zealand.

[f]Department of Crop Sciences, Institute of Agronomy, University of Natural Resources and Life Sciences, Vienna, Konrad-Lorenz-Strasse 24, 3430, Tulln, Austria.

[1]These authors contributed equally to this work.

*Corresponding author: jakob.santner@boku.ac.at

Table of Contents

1 Soil analysis 3

2 Fabrication of As^{III}-selective DGT gels (MSG) 5

3 LA-ICP-MS parameters 6

4 DGT LA-ICP-MS calibration standard preparation 7

5 DGT LA-ICP-MS method detection limits 9

6 O_2-optode fabrication and calibration 10

7 Area selection for solute flux quantification in rhizosphere and bulk soil 11

8 Exemplary visualization of fine-scale solute fluxes in the rhizosphere of *P. vittata* 13

References 14

1 Soil analysis

Soil characteristics were assessed using homogenized, air-dried and ≤2 mm-sieved soil. Organic carbon (C_{org}) and total nitrogen (N_t), $CaCO_3$ content, pseudo-total (*aqua regia* extractable) metal(loid) concentrations and pH were determined following Austrian standard methods (Blum et al., 1996). Soil water holding capacity (WHC) was determined by preparing a saturated soil paste according to Rhoades (1982). Electrical conductivity (EC) was measured using an EC meter (inoLab® Terminal 740, WTW, Weilheim, Germany) in the supernatant of a centrifuged (10 min at 15,300 × g) saturated soil paste. Dissolved organic carbon (DOC) was determined in a 1:10 (*w/v*) soil:laboratory water type 1 extract using a vario TOC cube (Elementar Analysensysteme GmbH, Langenselbold, Germany) after pre-treating 10 mL of extract with 18 µL of 10% (*w/w*) HCl to remove carbonates. Data on soil texture, clay content and cation exchange capacity (CEC) were taken from Fitz et al. (2003), as all analyzed soil properties were still in good agreement with previous measurements. Metal(loid) concentrations in *aqua regia* extractable soil fractions were measured using ICP-OES (Optima 8300, Perkin Elmer, Waltham, MA, USA), quadrupole ICP-MS (Elan 9000 DRCe, Perkin Elmer, Waltham, MA, USA) and sectorfield ICP-MS (ELEMENT XR, Thermo Fisher Scientific, Bremen, Germany) depending on the expected analyte concentrations. Internal standardization (^{72}Ge, ^{89}Y or ^{115}In) and external multi-point calibration (ICP multielemental standard VI, Certipur®, Merck) were applied. Certified (SPEX CertiPrep, New Jersey, USA) as well as in-house reference materials and blanks were analyzed throughout all analytical runs. Soil extracts, which were not immediately analyzed, were either frozen at -20 °C or acidified to 2% (*w/w*) HNO_3 and stored at 4 °C.

Table S1. Selected physico-chemical properties of the experimental soil (Forst99).

Parameter	Unit	Mean	±	SD[a]	Method
Textural class[b,c]	-	Sandy loam			ÖNORM L 1061
CEC[b,d]	mmol$_c$ kg^{-1}	334	±	n.a.	ÖNORM L 1086
WHC[e]	g kg^{-1}	706	±	39.0	saturated soil paste[f] (*n* = 4)
pH (H_2O)	-	7.96	±	0.04	ÖNORM L 1083 (*n* = 3)
pH ($CaCl_2$)	-	7.21	±	0.02	ÖNORM L 1083 (*n* = 3)
EC[g]	µS cm^{-1}	460	±	27.8	saturated soil paste[f] (*n* = 4)
$CaCO_3$	g kg^{-1}	135	±	15.5	ÖNORM L 1084 (*n* = 3)
C_{org}	g kg^{-1}	67.6	±	n.a.	ÖNORM L 1080 (*n* = 2)
N_t	g kg^{-1}	3.20	±	n.a.	dry combustion (*n* = 2)
DOC[h]	mg kg^{-1}	217	±	16.8	dry combustion (*n* = 4)
As	mg kg^{-1}	2080	±	18.1	ISO 11466: 1995 03 01 (*n* = 4)
P		1120	±	77.6	
Mn		1420	±	17.6	
Fe		53,700	±	530	

[a] Standard deviation
[b] Data taken from Fitz et al. (2003)
[c] According to IUSS Working Group WRB (2015)
[d] Cation exchange capacity
[e] Maximum water holding capacity
[f] According to Rhoades (1982)
[g] Electrical conductivity
[h] Dissolved organic carbon

2 Fabrication of As^{III}-selective DGT gels (MSG)

For the fabrication of ~100 µm-thin As^{III}-selective MSG gels in a polyurethane gel matrix, 3-mercaptopropyl-functionalized silica gel (M3; 200-400 mesh, Sigma-Aldrich) was ground to a bead size ≤10 µm using a high-frequency ball mill (~20 Hz for 2×5 min; MM 200, Retsch GmbH, Haan, Germany) with PTFE grinding equipment. Milled M3 powder was sonicated (RK510, Bandelin Sonorex, Berlin, Germany) in lab water type 1 (1:5, *w/v*) for 10 min. This suspension was topped up with a polyurethane (Hydromed-D4, AdvanSource biomaterials, MA, USA) solution (10 g Hydromed-D4 dissolved in 100 mL 1:10 (*w/v*) EtOH:lab water type 1) to reach 20 mL total volume. The resulting M3-containing gel cocktail was homogenized and knife-coated as a thin gel sheet following established protocols (Kreuzeder et al., 2013). Until usage, MSG were stored in the dark at 4 °C in polypropylene vials containing lab water type 1.

3 LA-ICP-MS parameters

Table S2. General LA-ICP-MS operating parameters over all analyses.

**Operating parameters**	
LA parameters (NWR193, ESI)	
Laser wavelength / nm	193
Pulse length / ns	4
Pulse frequency / Hz	20
Spot diameter / µm	150
Interline distance / µm	400
Scan speed / µm s^{-1}	250
Laser energy / %	30
Fluency / J cm^{-2}	1.6-3.0
Carrier gas	He
Carrier gas flow rate / L min^{-1}	0.9
ICP-MS parameters (NexION 350D, Perkin Elmer)	
RF power / W	1300
Gas	Ar
Nebulizer gas flow rate / L min^{-1}	1.0
Auxiliary gas flow rate / L min^{-1}	0.75
Plasma gas flow rate / L min^{-1}	15
Interface	Ni sampler + skimmer
Detector mode	Dual
Acquisition mode	Time resolved analysis
Measured isotopes	^{13}C, ^{24}Mg, ^{27}Al, ^{31}P, ^{44}Ca, ^{51}V, ^{55}Mn, ^{57}Fe, ^{63}Cu, ^{66}Zn, ^{75}As, ^{98}Mo, ^{114}Cd, ^{208}Pb
Integration time / s	0.453

4 DGT LA-ICP-MS calibration standard preparation

External As^{III} and As^{V} calibration standards were prepared by controlled loading of the solute species onto MSG and MBG using well-established DGT piston sampling procedures (Warnken et al., 2004; Kreuzeder et al., 2013; Hoefer et al., 2017). The P, Mn and Fe multi-element MBG standards were obtained from previous experiments (data not shown). Briefly, the ~100 µm thin resin gels were supported by a 0.4 mm thick spacer disc and then stacked with a 0.8 mm thick agarose-cross-linked polyacrylamide diffusive gel (Zhang and Davison, 1995) and a 0.14 mm thick hydrophilic polyether sulfone membrane (pore size: 0.45 µm; Sartorius AG, Göttingen, Germany) into DGT piston samplers with a exposure area of 3.14 cm^2 (DGT Research Ltd., Lancaster, UK). The samplers were immersed in quadruplicate into well (24 h)-equilibrated deployment solutions with either varying As^{III} (243, 3,638 and 6,985 µg L^{-1}) or As^{V} (102 and 610 µg L^{-1}) concentrations prepared from sodium salts of arsenous acid ($AsNaO_2$; ≥90%, Sigma-Aldrich) or arsenic acid ($Na_2HAsO_4 \cdot 7H_2O$; 98–102%, Alfa Aesar). A 0.001 mol L^{-1} $NaNO_3$ (>99%, Sigma-Aldrich) background and 0.05 mol L^{-1} MES buffer at pH ~5.6 was added. Grab samples were taken over the course of the DGT deployment period to determine As concentrations in solution. The DGT samplers were retrieved at varying time intervals derived from the targeted, theoretical gel loading, disassembled, and the resin gels were rinsed with lab water type 1. For each LA-ICP-MS calibration standard, one gel replicate was dried in a vacuum gel drier, cut to a 0.5 cm × 1 cm strip, and fixed onto a microscope slide for LA-ICP-MS analysis. Solute loading of the remaining resin gels (n = 3) was assessed by hot acid digestion using a 5:1 (*v*/*v*) mixture of HNO_3 (65%, *w*/*w*; EMPARTA, ACS, Merck) and H_2O_2 (30%, *w*/*w*; TraceSELECT Ultra, Fluka, Sigma-Aldrich) in either closed PFA-vessels on a hotplate at 160 °C for MSG or a high-pressure microwave system (Multiwave 3000, Anton Paar, Graz, Austria) for MBG (Kreuzeder et al., 2013). The MSG and MBG standard and blank gel digests were analyzed by sectorfield ICP-MS (ELEMENT XR, Thermo Fisher Scientific, Bremen, Germany) using ^{89}Y and ^{72}Ge for internal normalization, respectively. Certified

reference material (SPEX CertiPrep, New Jersey, USA) was measured periodically throughout each run with an average recovery of 99.3% ± 7.4%. The ICP-MS limit of detection (LOD) and limit of quantification (LOQ) were calculated as three times and ten times the standard deviation, respectively, of the corresponding MSG and MBG blank gels and were 2.37 and 7.89 ng cm^{-2} for As^{III} and 0.107 and 0.358 ng cm^{-2} for As^{V}, respectively. All measured gel loadings were above the calculated LOD and LOQ values.

5 DGT LA-ICP-MS method detection limits

The As, P, Mn and Fe blank levels in dried MSG and MBG were determined by LA-ICP-MS and used to calculate the respective As^{III}, As^{V}, P, Mn and Fe method detection limits (MDL = 3 × standard deviation of analyte signal on blank MSG or MBG). Analysis of P MDLs revealed substantial P contamination of the blank MBG surface during analysis of PV2, PQ2 and PQ3. Therefore, the average P MDL, excluding the contaminated blank gels, was used for the evaluation of the P background in the analysis of the respective MBG samples.

Table S3. Average (± SD), minimum (Min) and maximum (Max) method detection limits (MDLs) of As^{III}, As^{V}, P, Mn and Fe over all LA-ICP-MS runs (*n* = 5).

**Analyte**	**DGT gel**	**MDL (pg cm^{-2} s^{-1})**		
		**Average**	**Min**	**Max**
As^{III}	MSG	0.05 ± 0.02	0.03	0.07
As^{V}	MBG	0.01 ± 0.01	0.002	0.03
P	MBG	0.66 ± 0.46	0.43	1.34
Mn	MBG	0.05 ± 0.02	0.02	0.08
Fe	MBG	2.99 ± 0.38	2.66	3.65

6 O_2-optode fabrication and calibration

For fabrication of O_2-optodes, a 1%/1% (*w/w*) mixture of platinum(II)octaethylpor-phyrin (PtOEP) and Macrolex® yellow 10GN (LANXESS Deutschland GmbH, Cologne, Germany) was dissolved in 4% (*w/w*) polystyrene and knife-coated as a ~3 µm-thin dry sensor layer onto a double-sided adhesive polyester foil (Melinex® 506, 125 µm, Pütz GmbH + Co. Folien KG, Taunusstein, Germany) (Larsen et al., 2011). Calibration of O_2-optodes was performed using a modified Stern-Volmer equation according to Larsen et al. (2011). The calibrations were conducted in a transparent plastic container containing $Na_2S_2O_4$ at 10 g L^{-1} to determine 0% O_2 air saturation (R_0). Subsequently, the solution was exchanged with deionized water purged with N_2 (~2-3% O_2 air saturation) and the O_2 saturation was gradually increased using an aquarium air pump. O_2-optode images were taken every ~10% increase at 23 °C and ambient pressure. O_2 concentrations in solution were continuously determined using an oxymeter (HQ30D flexi, HACH, Loveland, CO, USA). Color ratiometric image calculations were performed as reported earlier (Larsen et al., 2011).

7 Area selection for solute flux quantification in rhizosphere and bulk soil

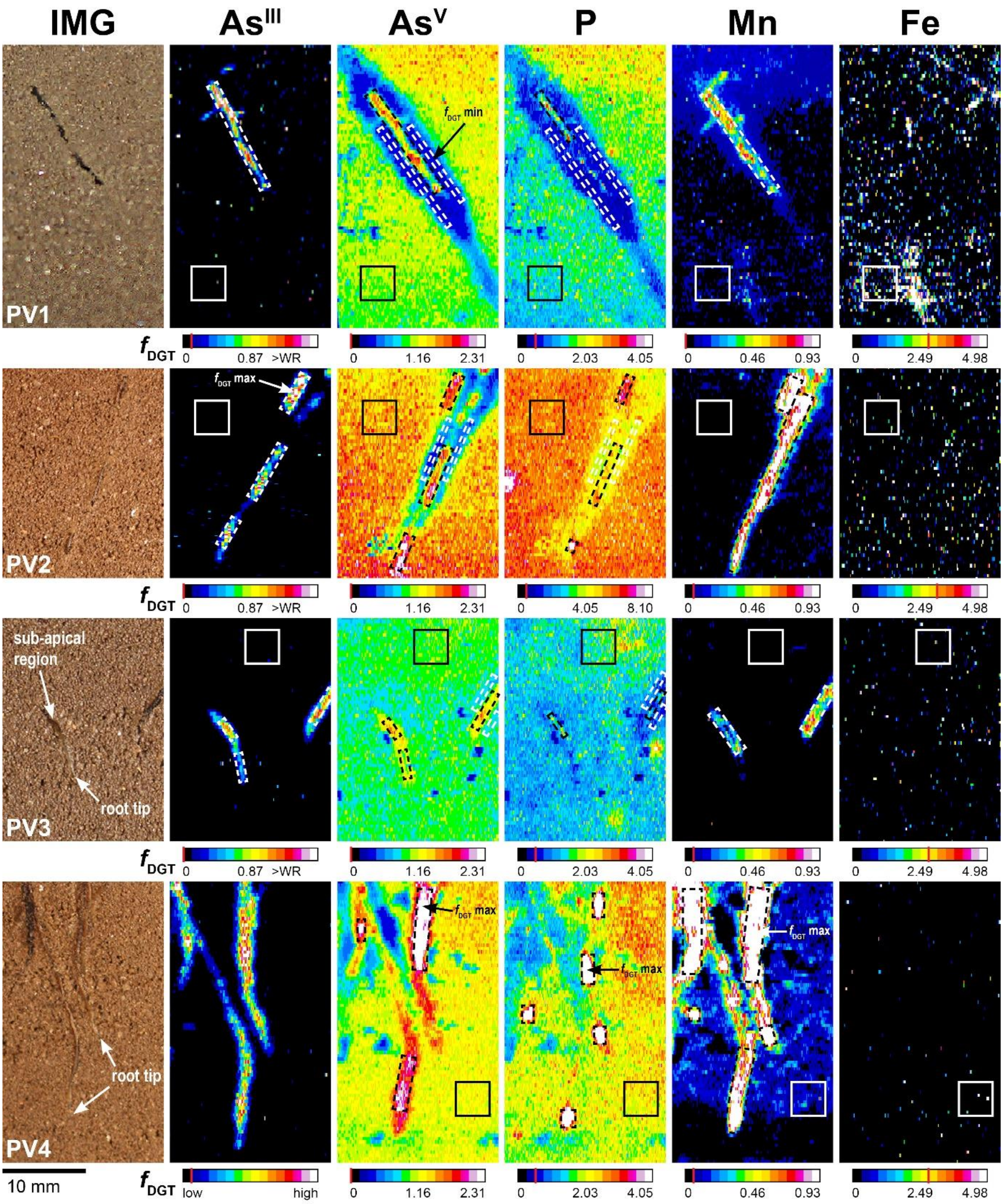

Figure S1. Solute images of As^{III}, As^{V}, P, Mn and Fe in the rhizosphere of PV showing the areas selected for solute flux quantification in rhizosphere (dashed rectangles) and bulk soil (solid rectangles).

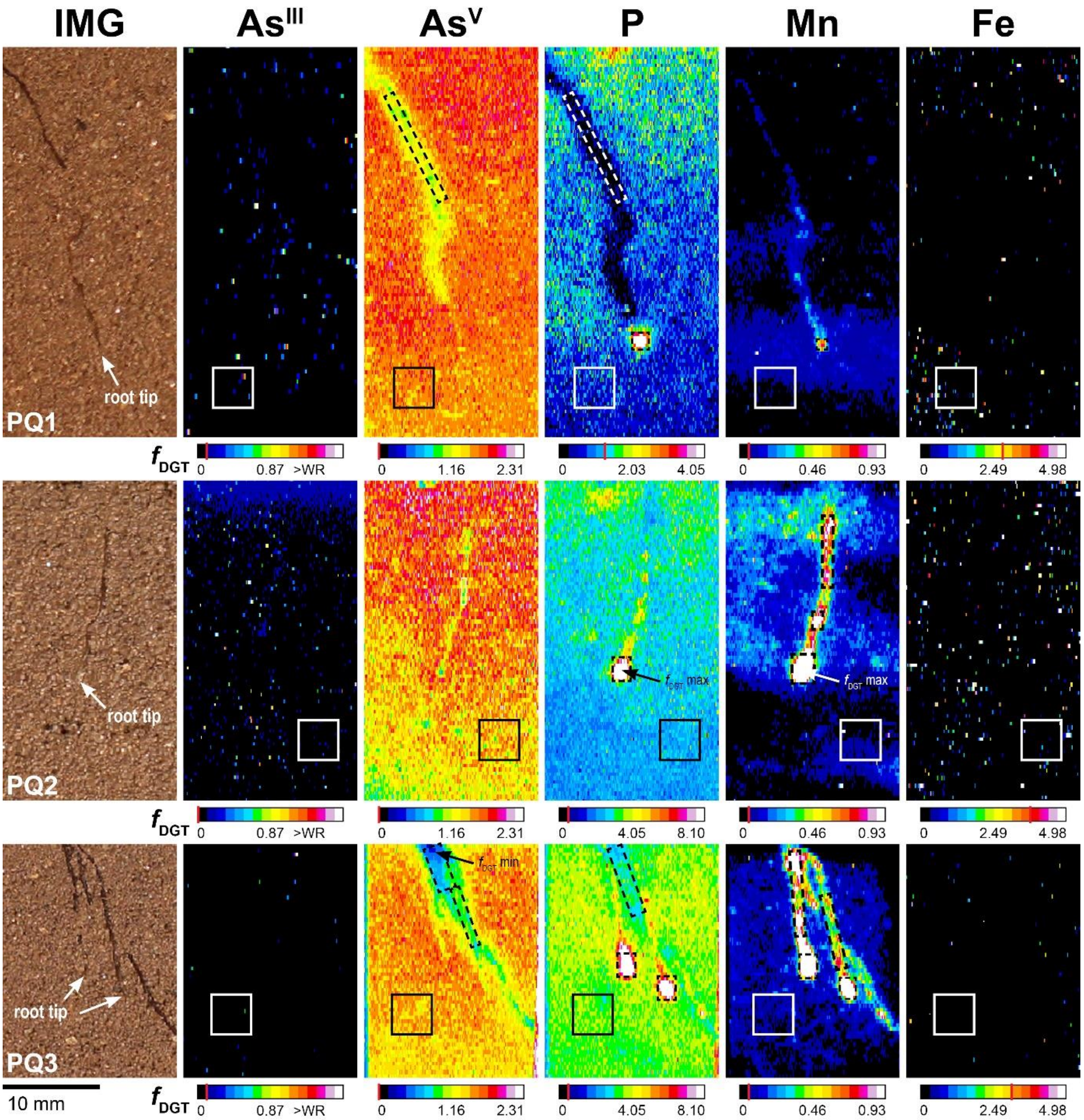

Figure S2. Solute images of As^{III}, As^{V}, P, Mn and Fe in the rhizosphere of PQ showing the areas selected for solute flux quantification in rhizosphere (dashed rectangles) and bulk soil (solid rectangles).

8 Exemplary visualization of fine-scale solute fluxes in the rhizosphere of *P. vittata*

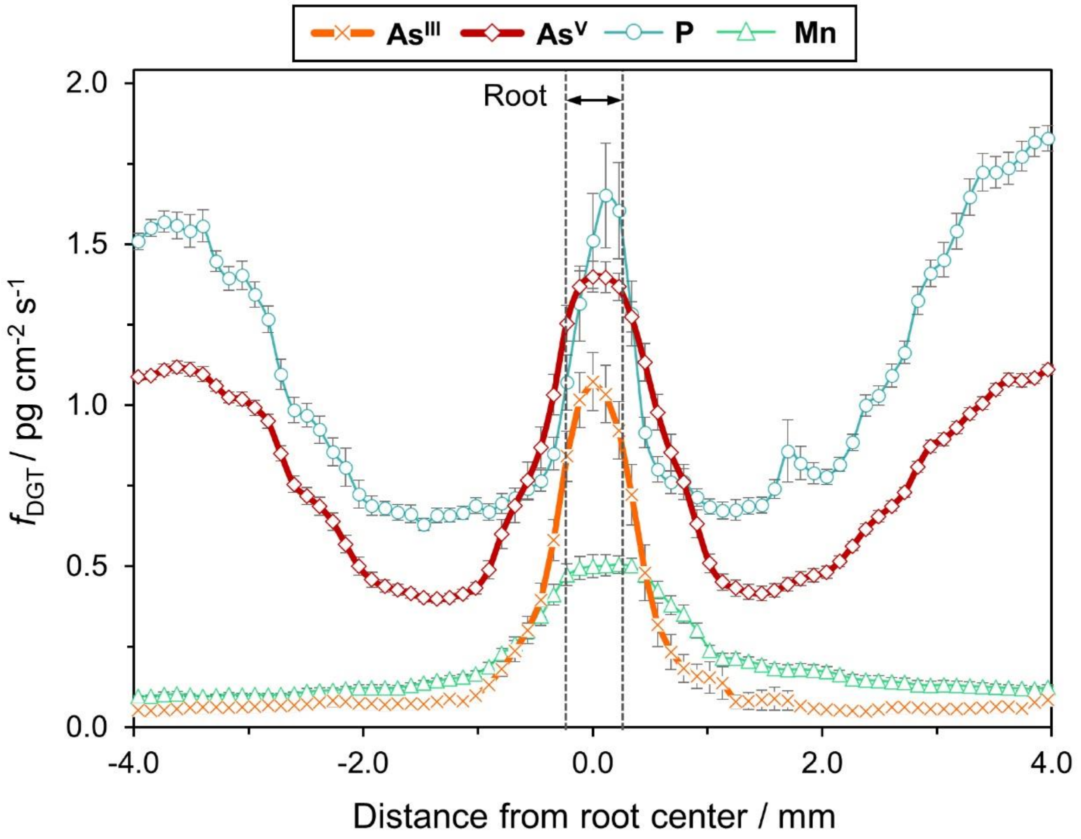

Figure S3. Average solute flux profiles of As^{III}, As^{V}, P and Mn shown as metal(loid) fluxes, f_{DGT}, in pg cm^{-2} s^{-1} across the soil-root interface of PV1. Each datapoint is the average of 32 parallel datapoints with a lateral distance of 113 µm obtained from 32 parallel lines which were extracted from the respective As^{III}, As^{V}, P and Mn solute images of PV1 shown in Fig. 3. The lines covered the whole root and were directed perpendicular to its axis with the line center corresponding to the root center, i.e. $x = 0$. The location of the PV1 root axis (average diameter of 0.5 mm) is depicted by the vertical dashed lines. Error bars display the standard error of the mean.

References

Blum, W.E.H., Spiegel, H., Wenzel, W.W., 1996. Bodenzustandsinventur, Konzeption und Durchführung. Bundesminsterium für Land und Forstwirtschaft und Bundesministerium für Wissenschaft; Verkehr und Kunst, Vienna.

Fitz, W.J., Wenzel, W.W., Zhang, H., Nurmi, J., Stipek, K., Fischerova, Z., Schweiger, P., Kollensperger, G., Ma, L.Q., Stingeder, G., 2003. Rhizosphere characteristics of the arsenic hyperaccumulator Pteris vittata L. and monitoring of phytoremoval efficiency. Environ. Sci. Technol. 37, 5008-5014.

Hoefer, C., Santner, J., Borisov, S.M., Wenzel, W.W., Puschenreiter, M., 2017. Integrating chemical imaging of cationic trace metal solutes and pH into a single hydrogel layer. Anal. Chim. Acta. 950, 88-97.

IUSS Working Group WRB, 2015. World reference base for soil resources 2014 (update 2015): International soil classification system for naming soils and creating legends for soil maps, World Soil Resources Reports. Food and Agriculture Organization of the United Nations (FAO), Rome.

Kreuzeder, A., Santner, J., Prohaska, T., Wenzel, W.W., 2013. Gel for simultaneous chemical imaging of anionic and cationic solutes using diffusive gradients in thin films. Anal. Chem. 85, 12028-12036.

Larsen, M., Borisov, S.M., Grunwald, B., Klimant, I., Glud, R.N., 2011. A simple and inexpensive high resolution color ratiometric planar optode imaging approach: application to oxygen and pH sensing. Limnol. Oceanogr. Methods 9, 348-360.

Rhoades, J.D., 1982. Soluble Salts. In: Page, A.L., Miller, R.H., Kenney, D.R. (Eds.), Methods of Soil Analysis. Part 2. Chemical and Microbiological Properties. American Society of Agronomy, Soil Science Society of America, Madison, WI, USA, 167-178.

Warnken, K.W., Zhang, H., Davison, W., 2004. Analysis of polyacrylamide gels for trace metals using diffusive gradients in thin films and laser ablation inductively coupled plasma mass spectrometry. Anal. Chem. 76, 6077-6084.

Zhang, H., Davison, W., 1995. Performance characteristics of diffusion gradients in thin films for the in situ measurement of trace metals in aqueous solution. Anal. Chem. 67, 3391-3400.
